# Editorial: Evolutionary and Integrative Approaches for Revealing Adaptive Mechanisms in Marine Animals Along Environmental Gradients

**DOI:** 10.3389/fphys.2020.00764

**Published:** 2020-07-14

**Authors:** Nelly Tremblay, Pierre U. Blier, Carlos Rosas

**Affiliations:** ^1^Shelf Sea System Ecology, Alfred-Wegener-Institut Helmholtz-Zentrum für Polar- und Meeresforschung, Helgoland, Germany; ^2^Département de Biologie, Université du Québec à Rimouski, Rimouski, QC, Canada; ^3^Unidad Multidisciplinaria de Docencia e Investigación, Facultad de Ciencias, Universidad Nacional Autónoma de México, Sisal, Mexico; ^4^Laboratorio Nacional de Resiliencia Costera, Consejo Nacional de Ciencia y Tecnología, Sisal, Mexico

**Keywords:** tropical latitudes, hypoxia, warming, ectotherms, osmotic, plasticity

This Research Topic was a result of the 5th Workshop of Ecophysiology: Marine animal resilience in a changing world, held in November 2017 in Sisal (Mexico). The participants highlighted several gaps of knowledge in the mechanisms underlying thermal, oxygen sensing, and osmotic adaptations, particularly in tropical and subtropical marine species. Special attention was thus called to studies contrasting environmental gradients in subtropical and tropical latitudes, including plankton, benthos, and nekton, to better understand their physiological adaptations to multiple stressors.

Plankton species must cross pronounced gradients of temperature, salinity, and oxygen, potentially showing broad ecophysiological plasticity. Harada et al. demonstrated intraspecific variation in thermal tolerance among three populations of the sub-tropical intertidal copepod *Tigriopus californicus* collected over a latitudinal thermal gradient (14 to 18°C) along the coast of California (USA) and acclimated to 20°C. During acute thermal exposure (>34°C for 1 h), the lower latitude population performed better in terms of survival, higher upper thermal limit, and ATP synthesis capacity. There was also a tight correlation between a decline in ATP synthesis capacity and higher upper thermal limits, which suggests a role for mitochondria in setting these limits and indicates that divergence of mitochondrial function is likely a component of adaptation across latitudinal thermal gradients.

Hypoxia tolerance of 10 dominant euphausiid species from Atlantic (sub-tropical), Pacific (temperate, tropical), and Polar regions, with or without exposure to Oxygen Minimum Zones (OMZs) was investigated by Tremblay et al. using the regulation index. This index assesses the regulation ability of aquatic organisms that do not present a clear critical oxygen partial pressure breaking point in their respiration pattern. Species that migrated vertically over long distances in shallow OMZ habitats were qualified as metabolic suppressors, whereas species associated with upwelling events in the neritic area expressed almost perfect oxyconformity. At *in situ* temperatures, polar and deep OMZ species displayed the highest degree of oxyregulation. Most of the studied Euphausiid species have evolved, at all latitudes, various respiratory strategies coupled with oxygen levels and temperatures experienced during their vertical migration, which may help to buffer substantial changes in their respective trophic ecosystems under climate change.

Intertidal and shallow-water benthic communities experience physical environmental variations along with daily and tidal cycles, which exposes animals to high environmental heterogeneity. Brahim et al. investigated heat tolerance plasticity in terms of laboratory acclimation and natural acclimatization of different populations of the tropical rocky-intertidal snail *Echinolittorina malaccana*. Four laboratory treatments (constant cool 22–23°C, warm cycle 25–45°C, constant extra cool 20°C, and extra-warm cycle 25–50°C) yielded similar capacities to acclimate and adjust thermal limits, but the populations differed in the temperature range over which they can make adjustments. This work supports that, irrespective of latitude, habitat heterogeneity drives thermal plasticity selection and should be considered in future studies seeking to assess ectothermic animals' response to environmental warming.

Nitric oxide (NO) could be essential for intertidal organisms, for the adjustment of mitochondrial respiration to local oxygen content. González et al. investigated how NO modulates the hypoxia tolerance of the widely distributed blue mussel *Mytilus edulis* under moderate, severe hypoxia and normoxia (1, 7, and 21 kPa *p*O_2_). Using live imaging techniques on excised gill filaments, the authors showed that NO accumulated under hypoxia, causing blood vessel dilatation after only 30 min of acute exposure. In a parallel measurement on the same tissue, cytochrome c oxidase activity increased in the transgression to moderate hypoxia (7 kPa) to later decrease at severe hypoxia (1 kPa), indicating a potential stabilizing effect of these accumulated NO at 1 kPa. As hypoxia tolerance varies with temperature, this study highlights the importance of laying out the mechanism defining the plasticity of hypoxia tolerance in a changing environment and the adaptability of this trait.

Ectotherms' ability to perform routine activities such as locomotion and fitness-related behaviors should be altered by increasing ocean temperatures. One understudied aspect of invertebrates is the evolution of the functions of contractile proteins in muscle tissue. Muscular mechanics are crucial physiological traits for prey capture and predator avoidance. In an extensive genome analysis of the Pacific white shrimp *Litopenaeus vannamei* myosin gene family, Zhang et al. identified a significant expansion of *Myo2* subfamilies in abdominal muscles, and high expression of *Myo2* during pleonal and pleopod muscle formation and development beginning at the zoea larval stage. This research represents a baseline to study the evolution of crustacean myosin proteins in different thermal habitats and related adaptations to temperature under climate change pressures.

Many invertebrates with direct development and benthic behavior have limited mobility, necessitating physiological and immunological capacities adapted to local or regional environments. Pascual et al. examined the influence of surface temperature, associated with seasonal upwelling, on the physiological and immunological conditions of the tropical species *Octopus maya*. Octopuses from cooler habitats (27°C) in the upwelling zone and transitional zone were in better condition; they expressed higher concentrations of hemocyanin and lower activities of an essential component of the immune system, phenoloxidase. Specimens captured in the warmer zone (>27°C) reflected immunological compensation mechanisms likely associated with metabolic stress that appeared to impair reproduction.

To further explore the connection between reproductive performance and temperature, López-Galindo et al. investigated the transcriptomic profiles of testes from thermally stressed (30°C) and unstressed (24°C) adult male *O. maya* before and after mating. Functional annotation and pathway mapping of the 1,881 differentially expressed transcripts revealed temperature impacts on processes involved in spermatogenesis, gamete generation, germ cell and spermatid development, response to stress, inflammatory response, and apoptosis. In particular, transcripts encoding genes linked to male infertility (sperm motility and spermatogenesis) were overexpressed in the stressed individuals, which was validated by quantitative real-time PCR. These essential genes could be under selective pressures at high temperatures to restore male fertility. The two *O. maya* studies highlight the importance of temperature on the physiological condition, metabolism, immune function, and life cycle that determine the stability and persistence of this endemic population.

In vertebrates, a gradient approach was used by Hunter-Manseau et al. to characterize mitochondrial phenotypic adjustments of the heart tissue of eight ray-finned fishes with thermal niches ranging from −1.9 to 30.1°C. When measured close to their optimal temperature, the enzymes that regulate fatty acid oxidation had higher relative activity in the cold-adapted fish species. These species also exhibited higher cytochrome c oxidase activity, compared to the other enzymes of the Electron Transport System. Thus, at different temperature regimes, selection can act on the mitochondrial organization (i.e., the relative abundance of key enzymes) rather than only tuning mitochondrial content.

Katayama et al. conceptualized the multiple roles of osmoregulating hormones in a comparative review using mudskipper gobies as a model, as these animals bridge the gap between aquatic and terrestrial life. In mudskippers, dehydration triggers angiotensin II secretion when the buccal cavity is dry, but it also produces corticosteroids and neurohypophysial hormones secreted during the escape from predators/conspecifics or in other stressful situations. The result of all these stimuli is a migration toward water bodies. By questioning the origin of the conserved central action of mineralocorticoid signaling in all vertebrates, this review provides a highly relevant benchmark in the context of climate change, to further scrutinize the hormonal regulation of habitat preferences.

Through this Research Topic, it was clear that compensatory processes occur in sub-tropical and tropical organisms faced with environmental stresses. Broad latitudinal comparisons, over multiple time scales of exposure (acute, chronic, or evolutionary), and different organization levels are urgently required to assess better both climate change effects on a global scale and potential evolution and adaptations. More significant strategic collaborations amongst research institutes that share the same coastline are required to achieve these comparisons.

## Author Contributions

All authors listed have made a substantial, direct and intellectual contribution to the work, and approved it for publication.

## Conflict of Interest

The authors declare that the research was conducted in the absence of any commercial or financial relationships that could be construed as a potential conflict of interest.

